# Host Factors Alter Effects of Angiopoietin-Like Protein 8 on Glucose Homeostasis in Diabetic Mice

**DOI:** 10.4236/jdm.2016.64029

**Published:** 2016-11-17

**Authors:** Sichen Liu, Emily M. Smith, Timothy H. King, Lindsey Glenn, Michelle Trevino, So Hyun Park, Yui Machida, Ciriaco Villaflor, Wojciech Grzesik, Margaret A. Morris, Yumi Imai, Jerry L. Nadler

**Affiliations:** 1 Eastern Virginia Medical School, Department of Internal Medicine, Strelitz Diabetes Center, Norfolk, VA, USA; 2 Department of Internal Medicine, Fraternal Order of Eagles Diabetes Research Center, The University of Iowa Carver College of Medicine, Iowa City, IA, USA

**Keywords:** Angiopoietin-Like Protein 8, Type 1 Diabetes, NOD, 12-Lipoxygenase, Beta Cells, Glucose Homeostasis

## Abstract

Recovery of functional beta cell mass offers a biological cure for type 1 diabetes. However, beta cell mass is difficult to regain once lost since the proliferation rate of beta cells after youth is very low. Angiopoietin like-protein 8 (ANGPTL8), a peptide that has a role in the regulation of lipoprotein lipase activity, was reported to increase beta cell proliferation in mice in 2013. Subsequent studies of human ANGPTL8 for short term (3 to 8 days) in non-diabetic mice showed little or no increase in beta cell proliferation. Here, we examined the effect of ANGPTL8 on glucose homeostasis in models that have not been examined previously. We expressed mouse ANGPTL8 using adenovirus in 2 mouse models of diabetes (streptozotocin and Non-Obese Diabetic (NOD) mice) over 2 weeks. Also, we tested ANGPTL8 in NOD mice deficient in leukocyte 12-lipoxygenase (12LO), an enzyme that contributes to insulitis and loss of beta cell function in NOD, in an effort to determine whether 12LO deficiency alters the response to ANGPTL8. Adenovirus-mediated expression of ANGPTL8 lowered blood glucose levels in streptozotocin treated mice without an increase in beta cell proliferation or serum insulin concentration. While ANGPTL8 did not reverse hyperglycemia in overtly hyperglycemic NOD mice or alter glucose homeostasis of non-diabetic NOD mice, ANGPTL8 reduced blood glucose levels in 12LOKO NOD mice. However, the lower glucose levels in 12LOKO NOD were not associated with higher serum insulin levels or beta cell proliferation. In summary, while mouse ANGPTL8 does not increase beta cell proliferation in NOD mice or streptozotocin treated mice in agreement with studies in non-diabetic mice, it lowers blood glucose levels in multiple low-dose streptozotocin induced diabetes and 12LO deficiency indicating that host factors influence the impact of ANGPTL8 on glucose homeostasis.

## 1. Introduction

Beta cell loss due to autoimmunity is the central pathology of Type 1 Diabetes (T1D). Although there has been progress in the treatment of T1D, including the development of the artificial pancreas and islet transplantation, the restoration of functional autologous beta cells offers a biological cure for T1D [1]. As T1D prevalence is rising in the United States and globally, there is a strong and urgent need for therapies that increase endogenous beta cell mass [1]. However, the low proliferation rate of beta cells (<1%) after young age in both humans and rodents poses as a major obstacle for the restoration of beta cell mass [2].

Angiopoietin-like protein 8/betatrophin (ANGPTL8) is a peptide that is primarily produced in the liver and adipose tissues. Hepatic expression of ANGPTL8 is up-regulated in rodents under an insulin resistant state, when beta cell mass increases [3]. In 2013, Yi *et al*. reported that ANGPTL8 is a highly potent beta cell specific trophic factor that increases beta cell proliferation up to 17-fold of control in ICR mice [3]. Also, overexpression of human ANGPTL8 in the liver, skeletal muscle (SK), and pancreas over 4 weeks increased beta cell proliferation in non-diabetic rats and mildly lowered glucose levels in rats treated by a single dose of streptozotocin (STZ) [4]. However, most of the follow-up studies have yielded either very modest or no increase in beta cell proliferation after the increased hepatic expression of human ANGPTL8 or administration of human ANGPTL8 peptide in non-diabetic mice [5] [6] [7] [8]. Therefore, the potential role of ANGPTL8 as a therapeutic entity to increase beta cell mass remains uncertain.

ANGPTL8 is now considered to have a primary role in modulating lipid metabolism rather than regulating beta cell proliferation [9] [10]. The peptide is proposed to inhibit lipoprotein lipase through the activation of ANGPTL3 in the heart and SK [9] [10]. As ANGPTL8 levels increase after feeding, the peptide may contribute to the reduction of triglycerides (TG) uptake into the heart and SK postprandially and augment allocation of TG to adipose tissues for storage [9]. In support of its role as a regulator of lipid metabolism, administration of ANGPTL8 and hepatic overexpression of ANGPTL8 raise serum TG levels in mice and rats, while serum TG levels are reduced in ANGPTL8 deficient mice [3] [4] [8] [11]. In humans, polymorphisms of ANGPTL8 are associated with alterations in serum LDL cholesterol, HDL cholesterol, and TG levels [10] [12] [13]. Thus, ANGPTL8 holds the potential to serve as a therapeutic target of dyslipidemia. Considering the high prevalence of dyslipidemia in diabetics including patients with T1D, it is of interest to determine how ANGPTL8 affects glucose homeostasis and serum lipid profiles in T1D models. Although there are several publications that tested ANGPTL8 in non-diabetic rodents [3] [5] [6] [7] [8], only one studied a diabetic model that utilized single dose STZ in rats [4].

Here, we tested ANGPTL8 modification of glucose homeostasis and serum TG levels in multiple low-dose STZ-treated mice and non-obese diabetic (NOD) mice. Additionally, to determine whether reduced islet inflammation modifies the response to ANGPTL8, we tested NOD mice deficient for pro-inflammatory enzyme leukocyte 12-lipoxygenase (12LO), which contributes to insulitis and loss of beta cell function in NOD mice [14] [15].

## 2. Methods

### Animal studies

Experiments were performed in accordance with the Institutional Animal Care and Use Committee guidelines of Eastern Virginia Medical School (EVMS). Two-month old male C57Bl/6J mice (BL6) and four-month old female were purchased from Jackson Laboratory, (Bar Harbor, ME). 12LO-deficient female NOD mice (12LOKO NOD) described previously [14] [15] were bred at EVMS. All mice were housed in 12:12 h light/dark cycle at 22°C and fed regular rodent chow *ad libitum* throughout the study. NOD mice were considered diabetic if blood glucose levels were over 300 mg/dl in two consecutive daily readings [14]. For a STZ-induced diabetes model, two-month old male BL6 were given STZ (0.75 mg/ml in 0.1M sodium citrate) subcutaneously at 50 μg/g body weight (BW) daily for 5 days. 2.5 weeks after the last dose of STZ, adenoviruses from Vector Biolabs (Philadelphia, PA) expressing either green fluorescent protein (Ad-GFP) or mouse ANGPTL8 (Ad- ANGPTL8) were delivered via lateral tail vein injection at 1 × 10^9^ plaque forming units (PFU) per mouse. Female NOD and 12LOKO NOD received 1 × 10^9^ plaque forming units (PFU) per mouse of Ad-GFP or Ad-ANGPTL8 at 4 months of age. Two weeks after adenovirus injection, mice received 0.01 ml/g BW of BrdU labeling reagent (Invitrogen, Camarillo, CA) intraperitoneally (i.p.), and were euthanized by carbon dioxide asphyxiation the next day. Cardiac blood was collected for serum analyses. The entire pancreas of each mouse was fixed in 10% formalin for histological analyses. Each liver was snap frozen in liquid nitrogen upon harvest and stored at −80°C until analyses.

### Glucose homeostasis

For glucose tolerance test (GTT), mice fasted overnight received glucose i.p. at 1.5 mg/g BW 10 days after adenovirus administration. For insulin tolerance test (ITT), mice fasted for 4 h from the morning received insulin lispro (Eli Lilly, Indianapolis, IN) i.p. at 0.75 mIU/g BW 7 days after adenovirus administration. Tail blood glucose was measured at indicated times using a One Touch Ultra hand-held glucometer (LifeScan Inc. Milpitas, CA).

### Colorimetric assays

Cardiac blood samples were centrifuged at 7000 RPM for 10 min at room temperature to obtain serum. TG (Stanbio Laboratories, Boerne, TX), *β*-hydroxybutyrate (Stanbio), non-esterified FA (Wako Chemicals, Richmond, VA), and mouse insulin levels (Mercodia, Winston Salem, NC) were measured in the serum according to manufacturers’ instructions.

### mRNA and qPCR

Total RNA from liver tissues (50 – 100 mg) was prepared using Qiagen RNeasy kit (Qiagen, Valencia, CA) according to the manufacturer’s protocol and reverse transcribed with iScript (Bio-Rad, Hercules, CA). Gene expressions were measured using ABI TaqMan commercial primers (Applied Biosystems, Foster City CA) and results were expressed taking beta actin as an internal standard.

### Histological analyses of the pancreas

Paraffin-embedded pancreatic sections were incubated with primary antibodies followed by visualization using fluorescent secondary antibodies as previously published [16]. Primary antibodies: rabbit anti-insulin at 1:500 (Abcam, Cambridge, MA) and rat anti-bromodeoxyuridine (BrdU) at 1:1000 (Abcam). Secondary Abs: Cy-2 coupled donkey anti-rat IgG antibody at 1:200 (Jackson Immuno Research, West Grove, PA); Cy-3 coupled donkey anti-rabbit IgG antibody at 1:200 (Jackson Immuno Research). Nuclei were visualized with 1 μg/ml 4’,6-diamidino-2-phenylindole (DAPI, Life Technologies, Carlsbad, CA). Images of pancreatic sections were recorded with Axio Observer Z.1 fluorescent microscope with Axio Vision Imaging Software v 4.7.1.0 (Carl Zeiss, Oberkochen, Germany). Total beta cell area was measured via summation of insulin positive area in a pancreatic section with maximum footprint for each mouse using Mosai X (scan mode: meander, autofocus per tile) in Axio Vision. BrdU positive nuclei were manually selected if 1) it was surrounded by insulin and 2) superimposed with DAPI to pick up a proliferating beta-cell. The number of BrdU positive nuclei was corrected for total beta cell area of each mouse.

### Statistics

Data are presented as mean ± SEM. Differences of numeric parameters between two groups were assessed with Student’s *t*-tests or Mann-Whitney test, and those for multiple group comparisons were assessed with one-way ANOVA (Tukey post test). *p* < 0.05 were considered significant.

## 3. Results

### Adenovirus-mediated ANGPTL8 expression in the liver improved hyperglycemia in streptozotocin treated mice

**Figure 1.**
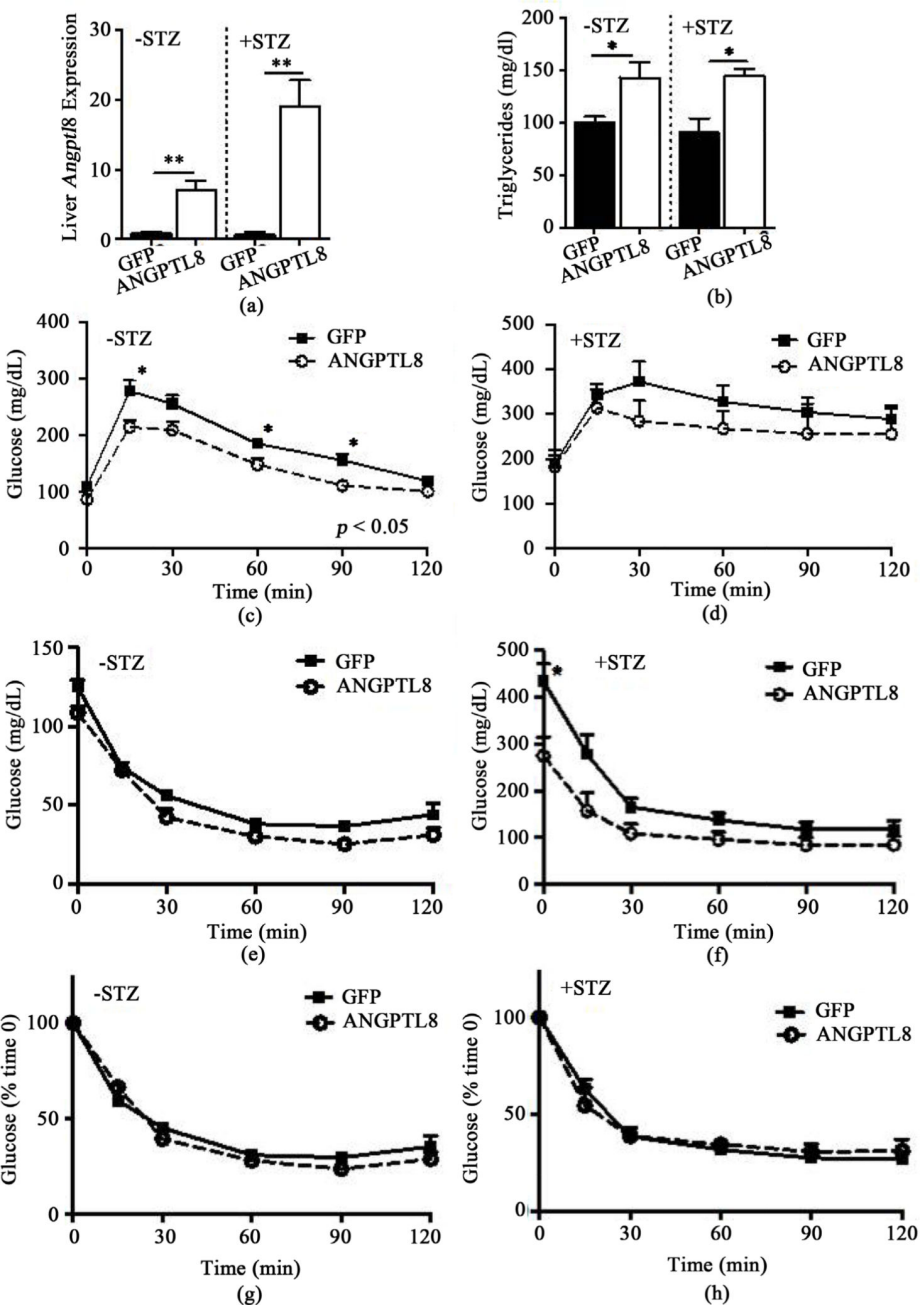
Glucose homeostasis of STZ pretreated and untreated mice with adenovirus mediated expression of ANGPTL8 or GFP in the liver. (a) The expression of ANGPTL8 was determined by qPCR in the liver of STZ pretreated (+STZ) or untreated (−STZ) mice followed by Ad-ANGPTL8 or Ad-GFP administration. Beta actin was used as an internal control. (b) Serum triglycerides at the time of harvest. GTT performed in overnight fasted −STD mice (c) and +STD mice (d) 10 days after Ad-ANGPTL8 or Ad-GFP administration. Glucose levels during ITT performed in 4 hour fasted –STD mice (e) and +STD mice (f) 7 days after Ad-ANGPTL8 or Ad-GFP administration. ITT expressed as % reduction in glucose in –STD mice (g) and +STD mice (h). Data are means ± SEM; *n* = 4 – 5 per group. *: *p* < 0.05, **: *p* < 0.01 between ANGPTL8 and GFP control groups. 2-Way ANOVA showed *p* < 0.05 for (c).

Ad-ANGPTL8 was administered to BL6 mice 2.5 weeks after the last dose of multiple low-dose STZ to determine how hepatic expression of ANGPTL8 affects glucose homeostasis and lipid profile in diabetic mice. qPCR of the liver harvested 2 weeks after Ad-ANGPTL8 injection confirmed that Ad-ANGPTL8 increased ANGPTL8 in the liver for both STZ (+STZ) and untreated (−STZ) mice (Figure 1(a), *p* < 0.01 for both groups). ANGPTL8 overexpression in the liver raised serum TG levels in both −STZ and +STZ mice to a similar extent (Figure 1(b), *p* < 0.05 for both groups). Ad- ANGPTL8 did not alter serum fatty acids or cholesterol levels in either −STZ or +STZ mice (data not shown).

**Figure 2.**
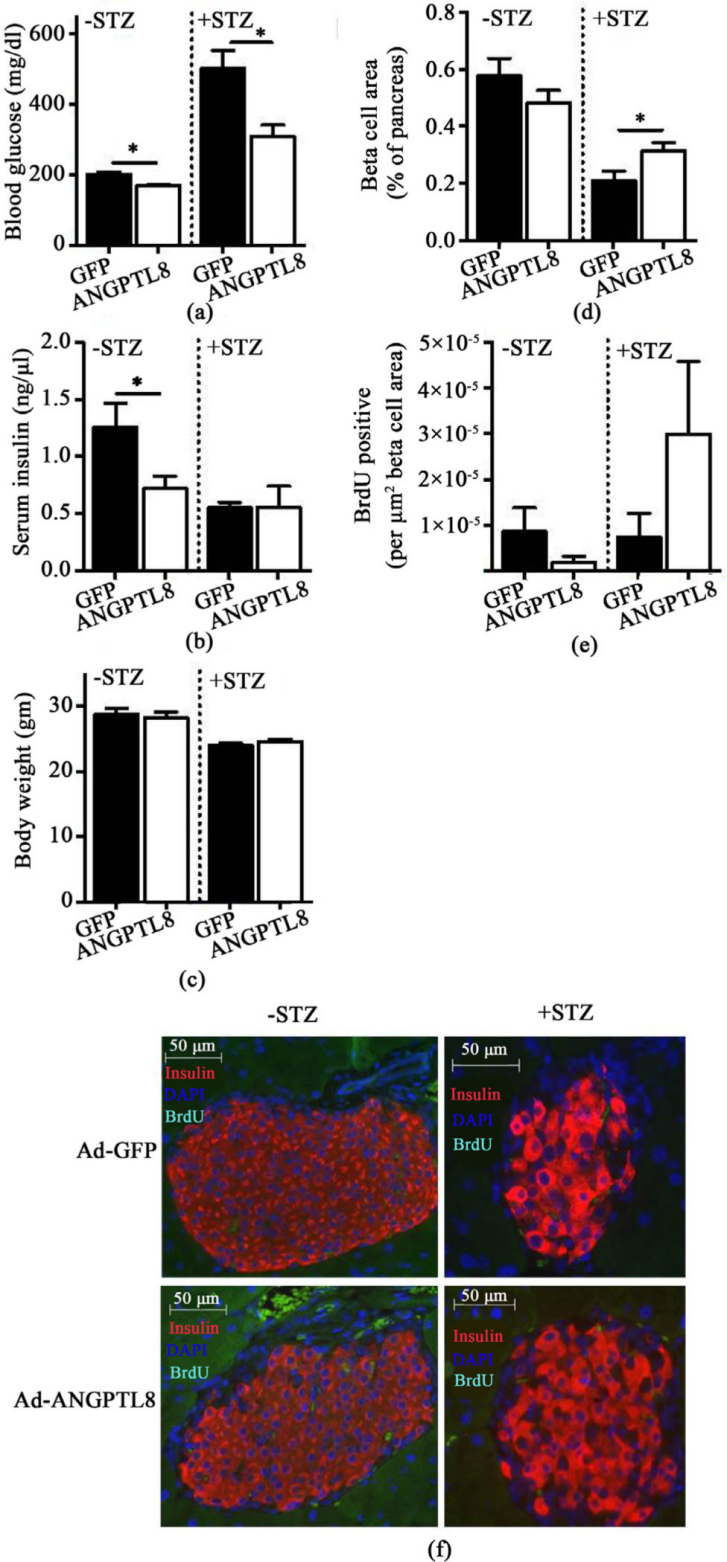
Serum chemistry and histology of STZ pretreated and untreated mice expressing ANGPTL8 or GFP in the liver. Blood glucose (a), serum insulin (b), and BW (c) on 14 days after Ad-ANGPTL8 or Ad-GFP treated mice that received STZ (+STZ) or untreated (−STZ) 14 days prior to adenovirus treatment. Beta cell area (d) and BrdU positive beta cells (e) compared between ANGPTL8 and GFP groups in −STZ and +STZ mice. (f) Representative pictures of –STZ and +STZ mice treated with Ad-GFP and Ad-ANGPTL8 immunostained with insulin and BrdU. Data are means ± SEM; *n* = 4 – 5 per group. *: *p* < 0.05.

We performed an ITT after a 4 hour fast on Day 7, and a GTT after an overnight fast on Day 10 after adenovirus administration. We did not observe any significant changes in weight between Ad-ANGPTL8 and Ad-GFP groups at the time of GTT or ITT (data not shown). ANGPTL8 overexpression improved glucose tolerance in −STZ mice (Figure 1(c), 2-Way ANOVA, *p* < 0.05), but not in +STZ mice (Figure 1(d)). During the ITT, baseline glucose levels were reduced in +STZ mice overexpressing ANGPTL8 but not in −STZ mice (Figure 1(e) and Figure 1(f)). Also, +STZ mice overexpressing ANGPTL8 showed a trend of reduced glucose levels during ITT (Figure 1(f), 2-Way ANOVA, *p* = 0.056). However, % reduction in glucose after ITT did not differ between Ad-ANGPTL8 and Ad-GFP in either −STZ or +STZ mice (Figure 1(g) and Figure 1(h)). We again noted that blood glucose levels were lower in the Ad-ANGPTL8 treated group at the time of harvest on Day 14 for both −STZ and +STZ mice, with a more prominent difference in the + STZ group (Figure 2(a), *p* < 0.05 for both groups). Interestingly, while serum insulin was lower in –STZ Ad-ANGPTL8 mice vs. Ad-GFP control (*p* < 0.05), while there was not a significant change in serum insulin levels for +STZ mice (Figure 2(b)). There was no difference in BW between Ad-GFP and Ad- ANGPTL8 treated mice in neither–STZ nor + STZ group (Figure 2(c)).

Although the initial publication demonstrated the robust increase in beta cell mass and beta cell proliferation [3], subsequent studies indicated that ANGPTL8 has limited or no effect on beta cell proliferation in non-diabetic mice [5] [7]. In agreement with the latest data, our −STZ BL6 mice did not show a difference in beta cell mass or BrdU incorporation in beta cells despite reduced glucose levels following ANGPTL8 overexpression (Figure 2(d), Figure 2(e), and Figure 2(f)). In contrast, beta cell mass was significantly increased following Ad-ANGPTL8 treatment in +STZ mice (*p* < 0.05), indicating that +STZ mice respond differently to ANGPTL8 compared with −STZ mice (Figure 2(d)). However, BrdU labeling of beta cells did not show a significant difference between ANGPTL8 and GFP groups in +STZ mice (Figure 2(e)). Collectively, ANGPTL8 expression in the liver resulted in reduced blood glucose levels in −STZ and +STZ mice without a clear indication of an increase in beta cell proliferation.

### Adenovirus-mediated ANGPTL8 expression in the liver had little effects on glucose homeostasis in NOD mice

**Figure 3.**
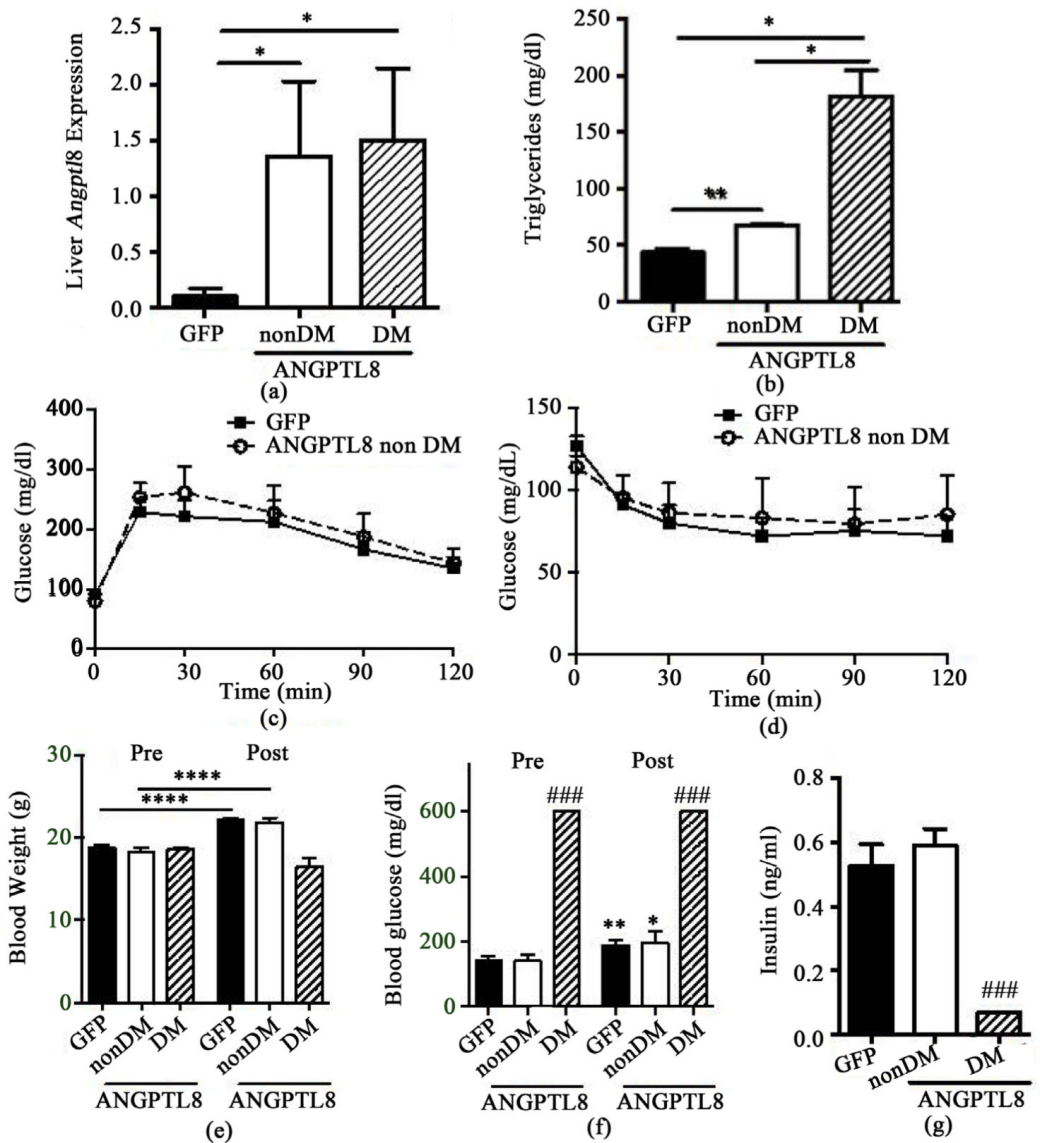
Glucose homeostasis of NOD mice that received Ad-ANGPTL8 or Ad-GFP. All diabetic (DM) NOD mice (n = 3) at the time of adenovirus administration received Ad-ANGPTL8, while non-diabetic (non-DM) mice were split into those receiving Ad-GFP (n = 10) or Ad-ANGPTL8DM (n = 5). Data are means ± SEM. (a) The expression of ANGPTL8 was determined by qPCR in the liver in Ad-ANGPTL8 (ANGPTL8) or Ad-GFP (GFP) treated NOD mice. Beta actin was used as an internal control; (b) Serum triglycerides at the time of harvest. *: *p* < 0.05, **: *p* < 0.01. GTT (c) and ITT (d) comparing non-DM NOD mice that received Ad- ANGPTL8 or Ad-GFP. Body weight (e) and blood glucose (f) prior to adenovirus administration (Pre) and 14 days after adenovirus administration (Post); (g) Serum insulin levels 14 days after adenovirus administration. *: *p* < 0.05. **: *p* < 0.01. ****: *p* < 0.0001 for paired t-test comparing pre and post. ###: *p* < 0.005 unpaired t-test compared with non-DM ANGPTL8 mice within the same time point.

We then tested whether adenovirus mediated expression of ANGPTL8 affects glucose homeostasis and serum TG levels in NOD mice. We obtained a clear increase in hepatic ANGPTL8 expression for both non-diabetic and diabetic NOD mice following injection of Ad-ANGPTL8 compared with mice receiving control Ad-GFP (Figure 3(a), *p* < 0.05). Furthermore, we observed a significant increase in serum TG with Ad- ANGPTL8, which was more pronounced in diabetic NOD compared with non-diabetic NOD mice (*p* < 0.01 to 0.05 as in Figure 3(b)). We did not observe any differences in GTT nor ITT between Ad-ANGPTL8 and Ad-GFP groups in non-diabetic NOD mice (Figure 3(c) and Figure 3(d)). During the 14-day period between adenovirus administration and the end of the experiment, non-diabetic mice continued to gain weight for both Ad-ANGPTL8 and Ad-GFP groups (*p* < 0.0001 for both groups, Figure 3(e)), while NOD mice that were diabetic prior to Ad-ANGPTL8 administration failed to gain weight (Figure 3(e)). Non-diabetic NOD mice injected with either Ad-ANGPTL8 or Ad-GFP showed similar increases in blood glucose levels by day 14, while diabetic NOD mice remained overtly diabetic (Figure 3(f)). Ad-ANGPTL8 did not alter serum insulin levels in non-diabetic NOD mice, and serum insulin levels were low in diabetic NOD mice treated with Ad-ANGPTL8 (Figure 3(g), *p* < 0.005 vs. non-diabetic NOD mice treated with ANGPTL8). Thus, mouse ANGPTL8 alone had no effects on glucose homeostasis of NOD mice.

### Adenovirus-mediated ANGPTL8 expression in the liver reduced blood glucose levels of 12 LO-deficient NOD mice

**Figure 4.**
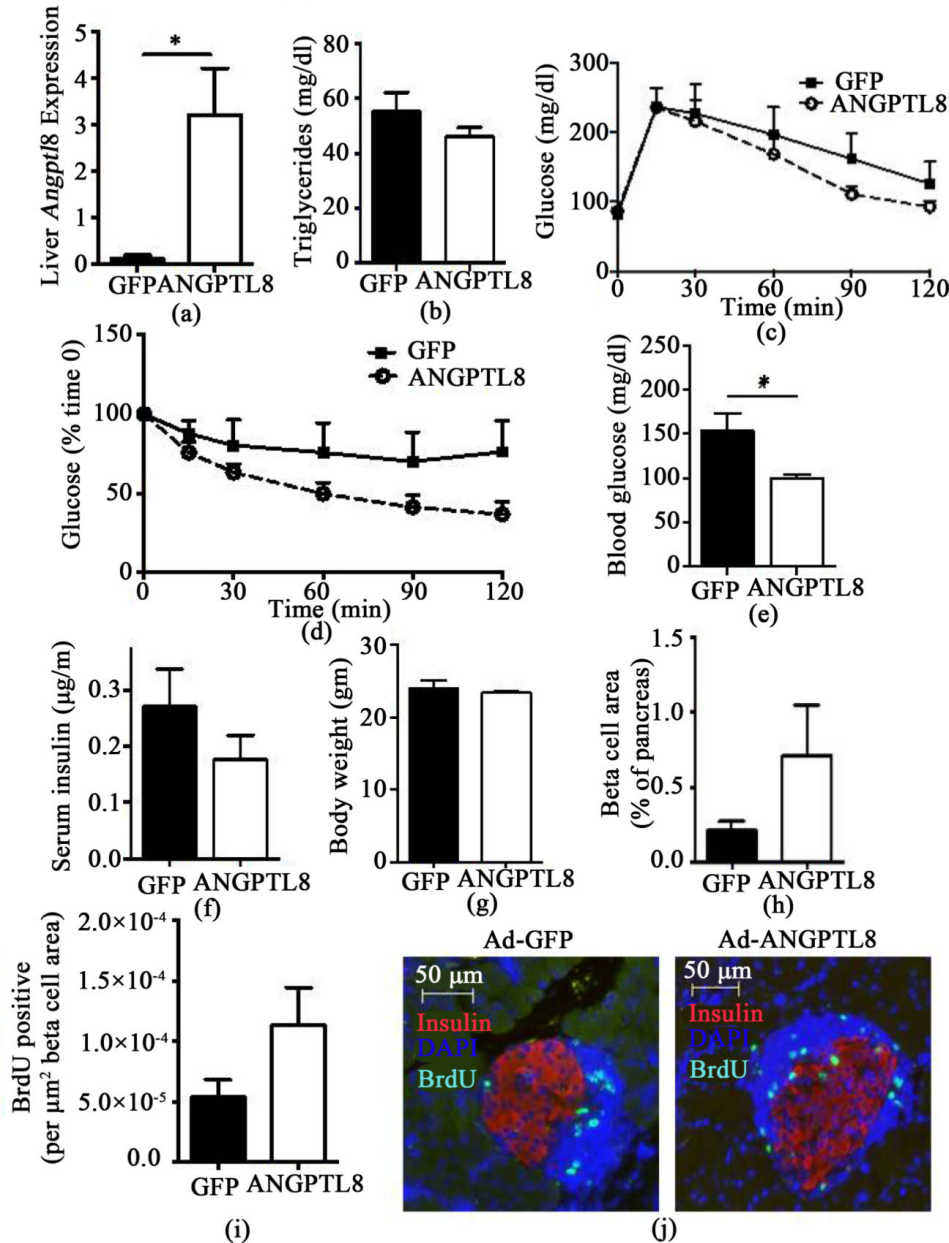
12LO deficient NOD mice treated with Ad-ANGPTL8 or Ad-GFP. (a) The expression of ANGPTL8 was determined by qPCR in the liver of Ad-ANGPTL8 (ANGPTL8) or Ad-GFP (GFP) treated 12LO deficient (12LOKO) NOD mice. Beta actin was used as an internal control; (b) Serum triglycerides at the time of harvest. GTT (c) and ITT (d) comparing 12LOKO NOD mice that received Ad-ANGPTL8 or Ad-GFP. Blood glucose (e) and serum insulin (f) after 4 hour fasting 13 days post adenovirus administration. BW (g) at the time of harvest. Beta cell area (h), BrdU positive beta cells (i), and representative image (j) compared between ANGPTL8 and GFP groups in 12LOKO NOD mice. Data are means ± SEM; *n* = 5 – 6 per group. *: *p* < 0.05.

Global deletion of 12LO in NOD mice (12LOKO NOD) prevents the development of diabetes [14] [15]. We used this model to test whether the absence of 12LO alters the response to ANGPTL8 overexpression in NOD mice. Although Ad-ANGPTL8 administration significantly increased hepatic ANGPTL8 compared with wild-type NOD mice (Figure 4(a), *p* < 0.05), 12LOKO NOD mice did not show an increase in serum TG levels (Figure 4(b)). Although GTT and ITT did not differ (Figure 4(c) and Figure 4(d)), it was interesting to note that Ad-ANGPTL8 reduced blood glucose levels in 12LOKO NOD mice on Day 13 after adenovirus administration (Figure 4(e), *p* < 0.05), but these changes were not associated with an increase in serum insulin levels (Figure 4(f)). There was no difference in BW between 12LOKO NOD mice treated with Ad- GFP and Ad-ANGPTL8 (Figure 4(g)). Beta cell area and BrdU incorporation into beta cells showed a large variation with higher average in Ad-ANGPTL8 treated mice, but did not show a statistically significant difference (Figure 4(h), Figure 4(i), and Figure 4(j)).

## 4. Discussion

Here, we report that adenovirus-mediated expression of mouse ANGPTL8 over 2 weeks alters glucose homeostasis and lipid profiles in 3 mouse models of diabetes. Our study demonstrated several novel points compared with previous studies [3] [4] [5] [6] [8]. We used mouse ANGPTL8, while previous studies tested human ANGPTL8 in rodents [3] [4] [5] [6] [8]. We tested 3 different models (multiple low-dose STZ, NOD, and 12LOKO NOD mice) to determine whether host factors affect responses to ANGPTL8. Except for one study that expressed ANGPTL8 in single dose STZ treated rats, previous studies were performed in non-diabetic rodents [3] [4] [5] [6] [8]. Also, the 14 day testing period we used was relatively long when compared with most of the studies that exposed mice to ANGPTL8 for 3 to 8 days [3] [5] [6] [8].

Our data in non-diabetic BL6 mice (−STZ) that received mouse Ad-ANGPTL8 agrees with the recent conclusions obtained from non-diabetic mice treated with human ANGPTL8 (Figure 2(d)); ANGPTL8 does not acutely increase beta cell proliferation in non-diabetic mice [5]. Although ANGPTL8 treated –STZ mice showed a small statistically significant reduction in blood glucose levels (Figure 2(a), *p* < 0.05), this was neither associated with an increase in beta cell mass nor higher serum insulin levels. The finding argues against beta cell proliferation as a factor supporting the small reduction in blood glucose after ANGPTL8 overexpression. The mechanism behind the lower glucose levels remains undetermined. Lower glucose levels in the face of reduced insulin levels implies better insulin sensitivity in insulin target tissues or reduced hepatic glucose production in ad-ANGPTL8 treated –STZ mice. Although the ITT did not show any significant differences (Figure 1(g)), the test may not be sensitive enough to detect small improvement in insulin sensitivity that accounts for small reduction in blood glucose levels. In comparison to –STZ mice, mouse ANGPTL8 expression after multiple low-dose STZ exposure resulted in sizable 37% reduction in blood glucose levels along with the increase in beta cell area compared with GFP control group (Figure 2(a) and Figure 2(c)). However, it should be noted that ANGPTL8 expression was not sufficient to normalize blood glucose levels or beta cell area in STZ induced diabetic mice. While it is plausible that ANGPTL8 affects beta cells exposed to STZ differently, the utility of ANGPTL8 in treating T1D is limited considering that it did not reduce blood glucose levels or increase insulin levels in either diabetic NOD nor non-diabetic NOD mice (Figure 3(f) and Figure 3(g)).

Furthermore, it is important to note that factors that increase beta cell proliferation in mice do not necessarily support the proliferation of human beta cells [17]. Although the action of ANGPTL8 was not directly tested, insulin receptor antagonist S961, which increases endogenous production of ANGPTL8, did not raise the proliferation of human beta cells transplanted into mice. This reduces the possibility that ANGPTL8 is a strong mitogen for human beta cells [18]. This is in contrast to two factors, SerpinB1 and a dual specificity tyrosine regulated kinase-1a (DYRK1A) inhibitor, that were recently reported to increase human beta cell proliferation [19] [20] [21] [22].

12LO is involved in arachidonic acid metabolism and produces 12-hydroxyeicosatetraenoic acid (12-HETE), which provokes inflammatory responses in beta cells and contributes to beta cell demise in T1D mouse models [23]. As 12LOKO NOD mice are protected against development of diabetes, the inhibition of 12LO has therapeutic potential for T1D [14] [15]. Interestingly, 12LOKO NOD did not show an increase in serum TG after ANGPTL8 overexpression (Figure 4(b)), a response persistently seen in –STZ mice, +STZ mice, and NOD mice. Blood glucose levels were also significantly lower in Ad-ANGPTL8 mice. Although not statistically significant, there was a trend of improved insulin tolerance and an increase in beta cell proliferation. These data indicate that the improvement in serum glucose can be multifactorial and reflecting the interplay between 12LO deficiency and ANGPTL8 elevation. Since a recent study indicated that ANGPTL8 may increase leukocyte infiltration into pancreatic islets [5], the lack of 12LO combined with ANGPTL8 may affect glucose homeostasis through the modulation of the immune system, which is known to affect glucose homeostasis both at the level of insulin sensitivity and in pancreatic beta cells [24] [25].

Although we observed the reduction in blood glucose levels by Ad-ANGPTL8 administration in –STZ BL6 mice, STZ BL6 mice, and 12LOKO NOD mice, the mechanism responsible for lower glucose levels remains unclear. This is a limitation of the current study. Further study should address both islet secretory function and insulin sensitivity in detail utilizing tests such as glucose-stimulated insulin secretion in vivo and in isolated islets, and euglycemic hyperinsulinemic cramp. Also, extending period of ANGPTL8 expression in the liver by transgenic or adenovirus-associated virus mediated expression may allow the detection of factors responsible for the alteration of glucose levels.

## 5. Conclusion

We confirmed that mouse ANGPTL8 is not a beta cell mitogen in non-diabetic mice. However, ANGPTL8 overexpression lowered glucose levels significantly in mice treated with low dose STZ and in 12LOKO NOD mice, indicating that ANGPTL8 may modulate glucose homeostasis, depending on host factors. The results suggest that reducing 12LO activity or expression could enhance the ability of beta cell mitogen to prevent or reverse T1D.

## Abbreviation List

ANGPTL8: Angiopoietin like-protein 8/betatrophin
NOD: Non-obese diabetic
T1D: Type 1 Diabetes
SK: Skeletal muscle
STZ: Streptozotocin
TG: Triglycerides
12LO: 12-lipoxygenase
12LOKO: 12-lipoxygenase knock-out mouse
BL6: C57BL/6 mice
Ad-GFP: GFP expressing control adenovirus
Ad-ANGPTL8: mouse ANGPTL8-expressing adenovirus
PFU: Plaque forming units
i.p.: intraperitoneal injection
GTT: Glucose tolerance test
ITT: Insulin tolerance test
+STZ: mice treated with STZ
−STZ: untreated control mice with respect to STZ administration
